# Issue Information

**DOI:** 10.1002/iid3.11

**Published:** 2014-07-26

## Aims and Scope

*Immunity, Inflammation and Disease* is a peer-reviewed, open access, interdisciplinary journal providing rapid publication of cutting-edge research across the broad fi eld of immunology. *Immunity, Inflammation and Disease* gives rapid consideration to papers in all areas of clinical and basic research. The journal welcomes original work that enhances the understanding of immunology in areas including cellular and molecular immunology, clinical immunology, allergy, immunochemistry, immunogenetics, cancer immunology, and many others.

Original research articles, methods papers, editorials and commentaries are published. Original research papers must report well-conducted research with conclusions supported by the data presented in the paper. *Immunity, Inflammation and Disease* considers for publication both papers submitted directly to the journal and those referred from a select group of prestigious journals published by Wiley Blackwell. For further information, please refer to our Author Guidelines available at http://onlinelibrary.wiley.com/journal/10.1002/(ISSN)2050-4527/homepage/ForAuthors.html

## Open Access and Copyright

*Immunity, Inflammation and Disease* is a Wiley Open Access journal, one of a series of peer-reviewed titles publishing quality research with speed and effi ciency. For further information, please visit the Wiley Open Access website at http://www.wileyopenaccess.com/view/index.html

All articles published by *Immunity, Inflammation and Disease* are fully open access: immediately freely available to read, download and share. Articles are published under the terms of the Creative Commons Attribution License which permits use, distribution and reproduction in any medium, provided the original work is properly cited.

Copyright on any research article in a journal published by *Immunity, Inflammation and Disease* is retained by the author(s). Authors grant Wiley a licence to publish the article and identify itself as the original publisher. Authors also grant any third party the right to use the article freely as long as its integrity is maintained and its original authors, citation details and publisher are identifi ed. Further information about open access license and copyright can be found in this website: http://www.wileyopenaccess.com/details/content/12f25db4c87/Copyright--License.html

## Disclaimer

The Publisher and Editors cannot be held responsible for errors or any consequences arising from the use of information contained in this journal; the views and opinions expressed do not necessarily refl ect those of the Publisher and Editors, neither does the publication of advertisements constitute any endorsements by the Publisher and Editors of the products advertised.

Wiley Open Access articles posted to repositories or websites are without warranty from Wiley of any kind, either express or implied, including, but not limited to, warranties of merchantability, fi tness for a particular purpose, or non-infringement. To the fullest extent permitted by law Wiley disclaims all liability for any loss or damage arising out of, or in connection with, the use of or inability to use the content.

## Editor-in-Chief

Dr. Marc Veldhoen

Babraham Institute, UK

Address correspondence to the Editorial Office: Email: IIDeditorial@wiley.com

## Editorial Board

Omid Akbari

University of Southern California, USA

Mark Coles

University of York, UK

Anselm Enders

The Australian National University, Australia

Jochen Huehn

Helmholz Centre for Infection Research, Germany

Stéphanie Hugues

University of Geneva, Switzerland

Igor Jurak

Harvard University, USA and University of Rijeka, Croatia

Ludger Klein

Ludwig Maximilian University of Munich, Germany

Jun Kunisawa

National Institute of Biomedical Innovation, Japan

Arian Laurence

Bethesda, MD, USA

Kathy McCoy

University of Bern, Switzerland

Osamu Takeuchi

Kyoto University, Japan

Hai Qi

Tsinghua University, China

Tim Radstake

University Medical Center Utrecht, Netherlands

Bruno Silva-Santos

University of Lisbon, Portugal

Mark Travis

University of Manchester, UK

David Withers

University of Birmingham, UK

